# Droplet flow cytometry for single-cell analysis

**DOI:** 10.1039/d1ra02636d

**Published:** 2021-06-14

**Authors:** Ming Li, Hangrui Liu, Siyuan Zhuang, Keisuke Goda

**Affiliations:** School of Engineering, Macquarie University Sydney NSW 2109 Australia ming.li@mq.edu.au; Biomolecular Discovery Research Centre, Macquarie University Sydney NSW 2109 Australia; Department of Physics and Astronomy, Macquarie University Sydney NSW 2109 Australia; Department of Chemistry, The University of Tokyo Tokyo 113-0033 Japan goda@chem.s.u-tokyo.ac.jp; Institute of Technological Sciences, Wuhan University 430072 Hubei PR China; Department of Bioengineering, University of California Los Angeles CA 90095 USA

## Abstract

The interrogation of single cells has revolutionised biology and medicine by providing crucial unparalleled insights into cell-to-cell heterogeneity. Flow cytometry (including fluorescence-activated cell sorting) is one of the most versatile and high-throughput approaches for single-cell analysis by detecting multiple fluorescence parameters of individual cells in aqueous suspension as they flow past through a focus of excitation lasers. However, this approach relies on the expression of cell surface and intracellular biomarkers, which inevitably lacks spatial and temporal phenotypes and activities of cells, such as secreted proteins, extracellular metabolite production, and proliferation. Droplet microfluidics has recently emerged as a powerful tool for the encapsulation and manipulation of thousands to millions of individual cells within pico-litre microdroplets. Integrating flow cytometry with microdroplet architectures surrounded by aqueous solutions (*e.g.*, water-in-oil-in-water (W/O/W) double emulsion and hydrogel droplets) opens avenues for new cellular assays linking cell phenotypes to genotypes at the single-cell level. In this review, we discuss the capabilities and applications of droplet flow cytometry (DFC). This unique technique uses standard commercially available flow cytometry instruments to characterise or select individual microdroplets containing single cells of interest. We explore current challenges associated with DFC and present our visions for future development.

## Abstract 2

This work reviews recent advances in the integration of emulsion microdroplets and flow cytometry technologies, so-called droplet flow cytometry (DFC), for high-throughput single-cell analysis.

## Graphical abstract

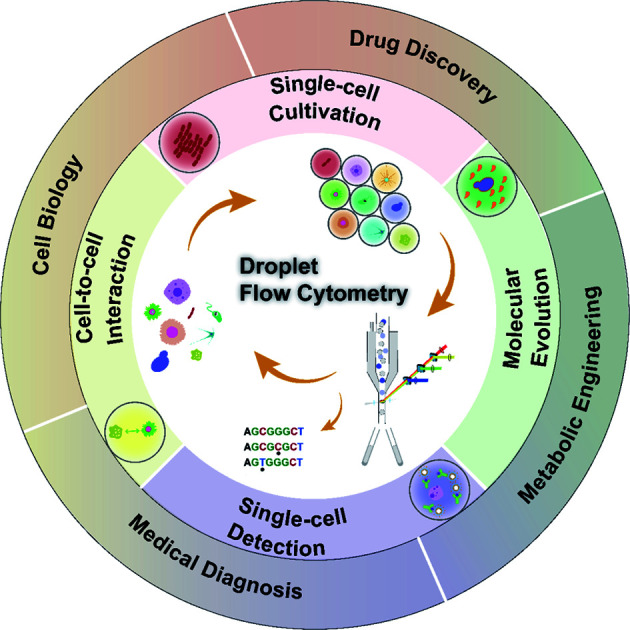

## 1. Introduction

Single-cell analysis plays an essential role in revealing the differences of individual cells in structure and function.^1,2^ The adaption of single-cell techniques has highlighted critical cell-to-cell heterogeneity, identified rare sub-populations of functional importance and discovered unique characteristics of individual cells.^3,4^ Flow cytometry (FC) has been widely used as a versatile and powerful technique to interrogate single cells, offering automated, quantitative, high-throughput and multi-parameter characterisation of cell phenotypes and activities.^5,6^ In this technique, individual cells suspended in a fluid flow are directed to pass through a laser beam one by one and then measured based on the resulting scatter or emission of light energy from fluorescence molecules. By adding cell sorting functionality, fluorescence-activated cell sorting (FACS, a specialised type of FC)^7,8^ can selectively isolate individual cells of particular interest from heterogeneous populations into separate containers (*e.g.*, tubes, well plates or other collection containers), allowing subsequent experiments on the selected cells. Single-cell analysis by FC (including FACS) has enabled significant advances in life and biomedical sciences, clinical diagnosis and drug development.^9–11^

However, FC-based single-cell analysis is exclusively limited to readouts from fluorescent molecules tagged either within the cells or on the cell surface. The attributes of individual cells are directly quantified and assessed by the light emitted by the fluorophore, which indicates the number of antibodies present within or on the cell. Unfortunately, this largely excludes important extracellular biomarkers and cell phenotypes and activities that are spatially and temporally important, such as secreted proteins, extracellular metabolite production, and proliferation. For example, the proteins secreted by cancer cells (*i.e.*, secretomes) could represent putative tumour biomarkers or therapeutic targets.^12^ Metabolites produced by industrial microorganisms have great values in nutrition, sustainable agriculture and healthcare sectors;^13^ real-time cell proliferation assays are essential in drug discovery and clinical evaluation.^14,15^ Therefore, there is a need to develop strategies that enable FC to make comprehensive measurements of cells, including extracellular, spatial and temporal biomarkers of biological, industrial and clinical significance.

Droplet microfluidics^16,17^ has emerged as an incredible tool in this effort, which can compartmentalise individual cells in pico-litre microdroplets containing a highly reproducible volume of a reaction mixture. Unlike conventional water-in-oil (W/O) droplets, two main architectures of droplets suspended in aqueous solutions are compatible with FC: water-in-oil-in-water (W/O/W)^18^ double emulsion (DE) droplets and hydrogel droplets.^19^ The pairing of FC and droplets allows a complete characterisation of individual cells beyond the conventional surface and intracellular biomarkers, featuring high throughput, sensitivity and dynamic range, and low cost. Moreover, it enables tandem genomic, epigenomic, or transcriptomic analyses on isolated cells,^20^ allowing integrative single-cell analysis techniques. This integrated approach has helped a wide range of discoveries, including directed evolution of enzymes and proteins,^21–24^ probing cellular heterogeneity in response to drug treatment,^25,26^ recognition of rare cells in microbial communities,^25,27–30^ determination of antibiotic resistance genes,^31,32^ and identification of biomarkers linked to diseases.^33,34^

Although fluorescence-activated droplet sorting (FADS)^35,36^ and other variants of the technique (*e.g.*, sequentially addressable dielectrophoretic array (SADA)^37,38^ and printed droplet microfluidics (PDM)^39^) allow on-chip screening and sorting of W/O droplets based on fluorescent readouts of in-droplet assays, the high complexity of custom devices and instruments involved limit their widespread adoptions.^35,40^ Moreover, they can only simultaneously measure 1 or 2 fluorescent parameters and requires relatively slow rates (*i.e.*, 0.1–2 kHz, two orders of magnitude slower than FACS) for high-accuracy sorting.^40^ Besides fluorescence, other optical detection techniques, such as adsorption spectroscopy^41^ and Raman spectroscopy,^42^ have been applied to characterise and select droplets encapsulated with individual cells in a microfluidic chip. However, these techniques suffer from low specificity (adsorption spectroscopy) and low sensitivity (Raman spectroscopy), making them unpopular in high-throughput droplet screening workflows.

**Fig. 1:**
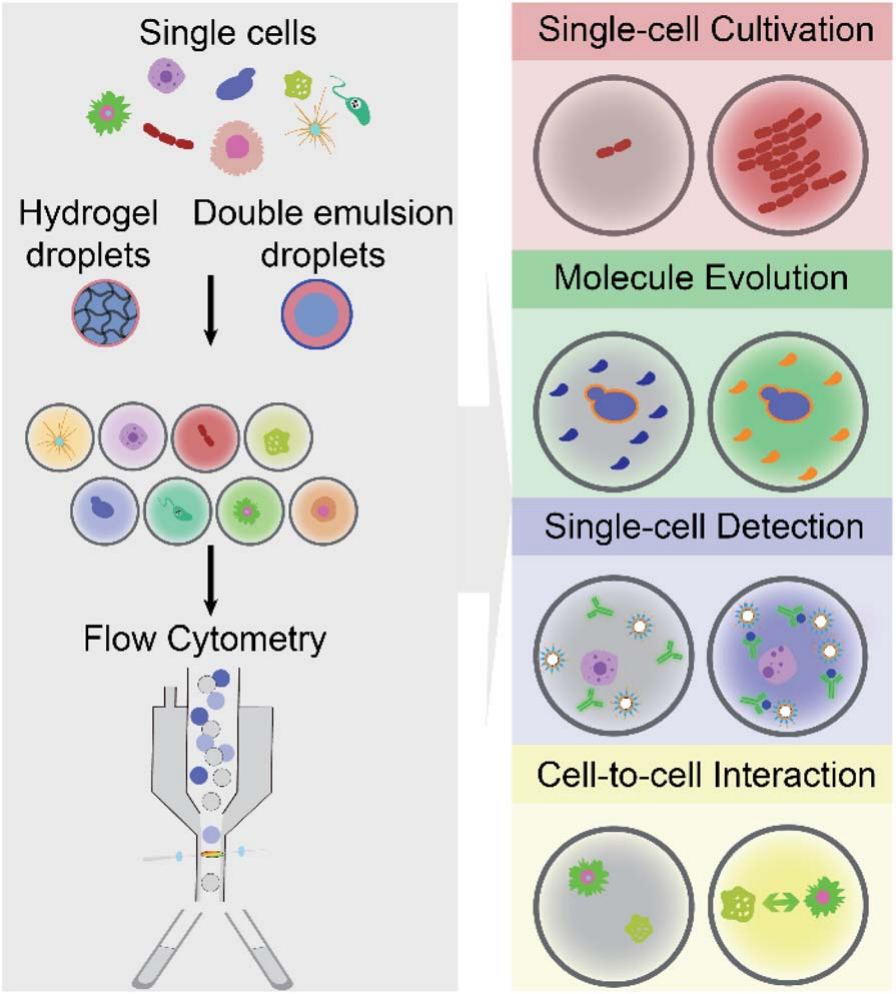
Droplet flow cytometry enables a wide range of high-throughput single-cell analysis, by integrating emulsion microdroplets (*e.g.*, water-in-oil-water double emulsion and hydrogel droplets) with commercially available flow cytometry.

The article reviews the current achievements in use of FC (including FACS) to screen and isolate microdroplets encapsulated with single cells of interest. It should be noted that we do not highlight advances in FADS and other techniques for screening and sorting of cell-laden W/O droplets on a microfluidic chip. As shown in Fig. 1, different types of single cells (*e.g.*, mammalian, bacterial and yeast cells) are encapsulated individually within microdroplets (either W/O/W DE droplets or hydrogel droplets), which can be screened and sorted by commercial FC machines, and further analysed at the downstream (*e.g.*, sequencing). This integrated technology, so called droplet flow cytometry (DFC), allows a variety of biochemical assays at the single-cell level, such as single-cell cultivation, molecular evolution, single-cell detection and cell-to-cell interactions, due to droplet monodispersity, cell compartmentalisation and high-throughput processing. Also, this opens avenues for broad applications in the fields of drug discovery, metabolic engineering, medical diagnosis and cell biology.

Specifically, this review is composed of the following sections. First, Section 2 introduces two main architectures of emulsion microdroplets compatible with FC, namely W/O/W and hydrogel droplets. Next, Section 3 summarised the main capabilities of integrating emulsion microdroplets and FC techniques (so-called DFC) for high-throughput single-cell analysis. Also, Section 4 discusses critical field applications of DFC. Furthermore, Section 5 discusses current challenges confronted by DFC with an eye towards future development. Finally, Section 6 concludes the review.

## 2. Architectures of emulsion microdroplets that are compatible with flow cytometry

An FC system is typically equipped with a fluidic system for cell handling, consisting of sheath fluid (*e.g.*, a buffered saline solution) to deliver and focus cells one by one in a narrow uniform stream. It enables cells to be equally illuminated, detected and measured as they pass through the laser intercept or interrogation point. Since FC requires cells to be suspended in an aqueous sheath fluid, it is not compatible with conventional W/O droplets having an insulating oil surrounding the aqueous core. Fortuitously, two main formats of droplets suspended in aqueous solutions, W/O/W DE droplets and hydrogel droplets, provide alternate droplet architectures compatible with FC. Individual cells can be encapsulated, cultured, subjected to biochemical assays in droplets; the cell-laden microdroplets can be detected as individual events by the cytometers and then screened or selected. Flow cytometric analysis of single-cell laden microdroplets allows a detailed study of cellular characteristics at the single-cell level by combining high throughput and rapid analysis with a relatively low reagent consumption and a high dynamic detection range.

### 2.1 Water-in-oil-in-water (W/O/W) double emulsion (DE) droplets

Conventional emulsion droplets can be either water-in-oil (W/O) or oil-in-water (O/W) single emulsions. There also can be more complex systems, such as W/O/W DE droplets, in which a W/O emulsion is dispersed in a second continuous aqueous phase.^43,44^ Therefore, W/O/W DEs can be viewed as two aqueous phases separated by a thin oil layer, stabilised by a monolayer of molecules of emulsifier or emulsifying agents at the interface between water and oil. Wettability plays a vital role in the types of emulsion droplets generated, as hydrophobic and hydrophilic surfaces are required to generate W/O and O/W emulsions, respectively.^45–47^ By selectively controlling the wettability of microchannels, microfluidic devices enabled a hierarchical generation of monodisperse W/O/W DEs encapsulated with individual cells.^47–50^

There are two main approaches to generate W/O/W DEs: the two-step batch method and the one-step continuous flow method using microfluidic devices.^51^ In the first approach, water phase and oil phase are mixed by stirring to form W/O droplets, which can then be encapsulated by another water phase *via* constantly adding more water under the stirring condition. However, DE droplets produced by this approach have large variations in size.^52^ The one-step continuous flow method is more widely used for single-cell encapsulation. DE production by this approach always requires a relatively high rate of oil phase flow to cut water phase with a low rate to generate W/O droplets, and thereafter a second aqueous flow with a much higher rate is used to cut the oil-water layer flow. Unlike the batch method, it ensures that microdroplets can be consecutively made with a uniform size.

In the DE, the aqueous internal phase can be composed of a reaction mixture (*e.g.*, substrates, buffers, fluorescent dyes and antibodies), cell suspensions or a gene library, providing a suitable environment for different cellular assays.^49,53,54^ The oil shell is bioinert and non-adhesive to hydrophilic activities and cells. The usage of fluorinated oil (*e.g.*, HFE-7500 and FC-40) acting as a selective barrier can reduce hydrophilic mass transfer and communication between DEs.^49,55,56^ The aqueous external phase allows the suspension of DEs in nutrient medium for long-term cell culture and the compatibility with commercial cytometers for high-throughput assays^18,49,57,58^ (with the sorting speed >10^7^ events per hour^31,32,56^).

The integration of FACS and W/O/W DE droplets have already found various applications, such as analysis of cell-secreted molecules and enzymatic turnover. Moreover, the isolated cells from heterogeneous populations allow downstream bioinformatics analysis (*e.g.*, tandem genomic, epigenomic and transcriptomic analyses) of the same target, revealing the biological functions of massive genetic pathways.^48–50^

### 2.2 Hydrogel droplets

Hydrogels are three-dimensional cross-linked hydrophilic polymer networks that are capable of swelling or de-swelling reversibly in water. By using monomer or polymer solutions as the aqueous dispersed phase and oil with a surfactant as the continuous phase, hydrogel droplets in oil phase can rapidly generate by either laboratory-customised 2D microfluidic devices^59^ or 3D-printed microdroplet generators^60^ for various biomedical assays. Unlike W/O droplets, the generated hydrogel droplets can be transferred from an oil suspension into an aqueous solution (*e.g.*, buffer or cell culture medium) after gelation for use in FC. The hydrogel microdroplets can offer a biologically friendly environment (mimic the extracellular environment by a three-dimensional matrix for cell culture). A microcapsule remains the activities of living cells^61–65^ and their genes,^66–69^ secreted proteins^70–73^ and other ingredients (*e.g.*, drugs^74–77^). Due to their biocompatibility, mechanical and chemical stability in aqueous media, sufficient permeability of porous structure and tunable polymer properties, hydrogels (both natural and synthetic) have been widely used in droplet microfluidics for single-cell assays.

Natural hydrogels used for emulsion microdroplets are either polysaccharides (*e.g.*, agarose, alginate, and chitosan) or proteins (*e.g.*, collagen and gelatin). For example, agarose is a linear neutral polysaccharide extracted from specific red seaweed, which undergoes sol–gel transition depending on temperature.^78^ Alginate is an anionic polysaccharide typically obtained from brown seaweed and bacteria, which chelates with alkaline earth metal ions, such as calcium (Ca^2+^), barium (Ba^2+^) and strontium (Sr^2+^) to form hydrogels. Gelatin is a protein derived from collagen, gels *via* a cold-setting mechanism and remains gel-like upon heating up to physiological temperature. It allows the liquid form to remain during the cultivation period of cells (*i.e.*, at 20–27 °C) and cell recovery under mild conditions (*i.e.*, at 35 °C) without cell damage.^79^

Besides, synthetic hydrogels, such as poly(ethylene glycol), poly(acrylic acid), poly(vinyl alcohol), poly(acrylamide) and their derivatives, offer desirable, controllable and reproducible properties (*e.g.*, molecular weights, structures and crosslinking density), which translate into tunable chemical, mechanical, and degradation properties of hydrogels. Typically, the aqueous monomer solutions are mixed with initiator and cross-linker, followed by polymerisation or crosslinking *via* different methods, such as photoirradiation. For example, poly(ethylene glycol) (PEG) can be chemically modified to generate ultraviolet (UV)-sensitive PEG-diacrylate that allows photoinitiated polymerisation.^80,81^

## 3. Capabilities

By encapsulating individual cells from a heterogeneous population within monodisperse microdroplets (*e.g.*, W/O/W DE and hydrogel droplets) and analysing cell-laden microdroplets as individual events by FC (including FACS), DFC has enabled new functionalities and diversified applications for single-cell analysis. This section aims to summarise and discuss four main capabilities of DFC, namely single-cell cultivation, molecular evolution, single-cell detection, and cell-to-cell interaction (see Fig. 1), which allow a broad range of applications in biological, biomedical and environmental fields.

### 3.1 Single-cell cultivation

**Fig. 2:**
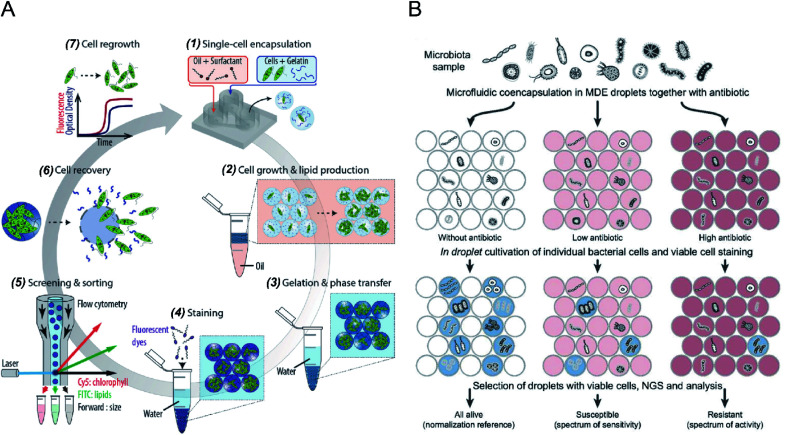
Droplet flow cytometry for single-cell cultivation. (A) Schematic workflow of screening and selecting microalgal cells with high biomass and lipid production rate. (1) Encapsulation of single microalgal cells within gelatin droplets, (2) cultivation and metabolite accumulation of single cells, (3) transfer of cell-laden hydrogel droplets from oil into an aqueous phase after gelation, (4) staining of target metabolites in hydrogel beads, (5) screening and sorting of hydrogel beads containing a high level of metabolites, (6) recovery of cells from the hydrogel beads, (7) regrowth of released cells, which can be reintroduced into iterative selection. Adapted with permission from ref. 79. Copyright 2018 John Wiley & Sons, Inc. (B) Schematic diagram of the activity/sensitivity spectrum assessment of a highly heterogeneous bacteria population (microbiota) using DEs. Individual cells from microbital samples were encapsulated in DEs together with different concentrations of the antibiotic, amicoumacin A (Ami). After cultivation, DEs were stained for metabolic activity, selected by FACS, and analysed by next-generation sequencing (NGS) and bioinformatics. Adapted with permission from ref. 31. Copyright 2018 National Academy of Sciences.

Microdroplets can act as miniaturised bioreactors to facilitate cell-based biological studies. This is because they mimic the micro-scale environment where biochemical events transpire and significantly enhance the efficiency and sensitivity of high-throughput cellular studies by reducing reagent consumption and improving detection signals.^82,83^ Flow cytometric analysis of single cells encapsulated in microdroplets was reported as a powerful tool for high-throughput screening of cell growth kinetics and metabolite accumulation.^31,32,56^ Y. Zhang *et al.*^56^ proposed flow cytometric characterisation of bacterial growth in DE droplets acting as programmable bioreactors. Unlike single emulsion droplets that are incapable of continuous supply of nutrient molecules, the oil shell in the DEs has demonstrated the capability to allow selective transport of small nutrient molecules and chemical inducers into the aqueous core. This study demonstrated the capability of tracking the growth of individual bacterial cells both qualitatively and quantitatively, where DEs enable more control over microbial cell growth conditions. Moreover, it shows the potential of using DEs for the study of complex biological processes requiring communication with external environments. M. Li *et al.*^79^ quantitatively tracked the growth and metabolite (*i.e.*, chlorophyll and lipid) accumulation of two microalgal species, *Euglena gracilis* (*E. gracilis*) and *Chlamydomonas reinhardtii* (*C. reinhardtii*), in gelatin hydrogel microdroplets using FACS (see Fig. 2A). The porous hydrogel structures enabled the infusion of fluorescent reporter molecules into the hydrogel matrices, and cells exhibiting desired properties (*e.g.*, high biomass and metabolite production) can be recovered after sorting for re-culture. In comparison to previous works, this research enables quantitative tracking of not only the growth of individual cells but also their metabolite production. The proposed technology can be easily integrated into directed cellular evolution pipelines for microbial strain improvement.

The integration of emulsion microdroplets with FACS has also enabled antibiotic susceptibility testing, the discovery of antibiotic resistance genes and the quantification of microbiome diversity. Y. J. Eun *et al.*^84^ encapsulated *E. coli* cells in agarose droplets and determined the minimum inhibitory concentration (MIC) of rifampicin using FACS. Spontaneous mutants resistant to antibiotic were isolated and further characterized by DNA sequencing, which identified β-subunit of RNA polymerase, RpoB, as the target. S. S. Terekhov *et al.*^31^ reported the culture of individual cells from a heterogeneous bacterial population in DE droplets to investigate the efficacy of an antibiotic, amicoumacin A (Ami), toward different microbiomes (see Fig. 2B). Unlike bulk cultures where fast-growing species can quickly dominate a population, single-cell cultivation in droplet compartments allows researchers to assess minor microbiota species representing <0.1% of the initial population. Later, potential antibiotic producers were selected using FACS, and a unique mechanism of self-resistance was discovered using proteomics and heterologous expression. This outcome shows that DFC could be efficiently applied to analyse complex microbial communities, and potentially expanded to functional profiling of eukaryotic cells.

In addition to microorganisms (*e.g*., bacterial and microalgal cells), mammalian cells have been successfully encapsulated and cultured in DE droplets for high-throughput screening. K. K. Brower *et al.*^18^ performed the phenotyping of individual cells from four mammalian cells lines, including mouse embryonic stem cells, human T lymphocytes, mouse macrophage cells, human embryonic kidney cells, and mouse embryonic fibroblasts having different morphologies and sizes (5–20 μm), in DEs (with an inner diameter of 35 μm). Using a live-cell labelling dye, Calcein Blue AM, as an indicator of cell-laden DEs, FACS measurement results successfully discriminated empty droplets from those containing cells, which showed good agreement with theoretical predictions by Poisson distribution. This high-throughput screening of DEs encapsulated with different types of mammalian cells opens up new avenues for various biomedical applications, such as disease diagnostics, drug discovery, and rare mutation detection.

### 3.2 Molecular evolution

Microbial production of industrial enzymes and other value-added bioproducts is attractive due to their advantages compared to conventional chemical syntheses, such as sustainability, reduction in environmental pollution, and cost-effectiveness.^6^ Although agar plate screening and microtiter plate assays enable automatic screening of enzymatic activities, they are costly and low-throughput, limiting the number of strain variants that can be evaluated (typically in the range of 10^3^–10^4^).^53,54,85^ Single-cell FACS has already demonstrated its capability to select individual cells exhibiting desired properties from a heterogeneous cell population, significantly increasing the screening throughput (with sorting speed >10^7^ events per hour).^86^ However, FACS for enzyme selection is limited to the cases where signalling molecules have restricted diffusion out of the cells or can be entrapped on the cell surface or antibody-conjugated microbeads. This limitation can be overcome by encapsulating single cells within emulsion microdroplets, which restrict the diffusion of products by compartmentalization, and the fluorescence intensity of the whole droplet can be detected by FC, which is also accurately correlated with the activity of encapsulated individual cells.

**Fig. 3:**
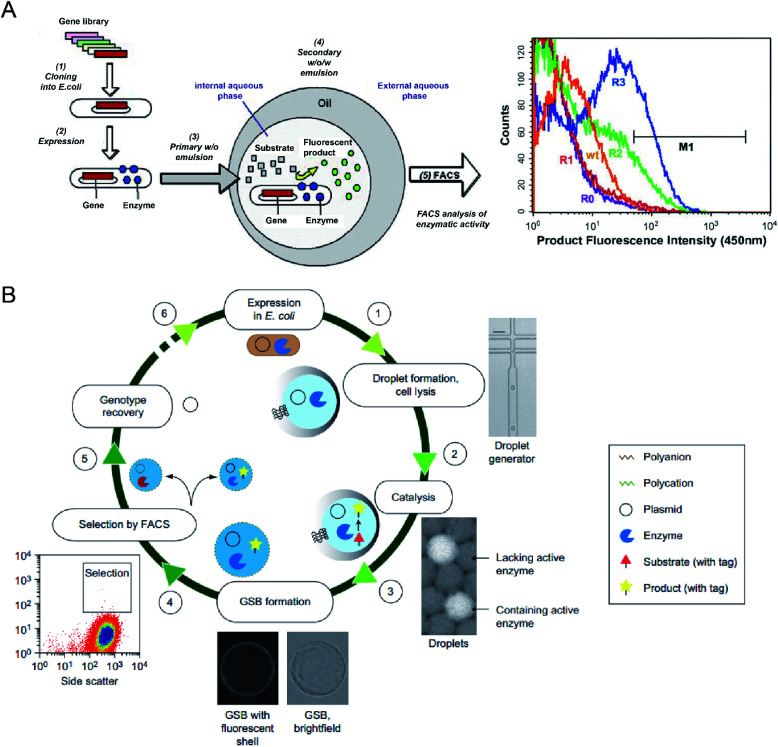
Droplet flow cytometry for molecular evolution. (A) Schematic workflow of directed enzyme evolution by screening and selecting positive DE droplets *via* FACS. Left: (1) a variant gene library was transformed and cloned into *E. coli*, (2) the encoded proteins translated within *E. coli*, (3) encapsulation of single cells in the W/O emulsion droplets, (4) addition of fluorogenic substrates through the oil phase and the formation of W/O/W DE droplets. Cells with functional enzymes can convert the non-fluorescence substrates into fluorescent products entrapped in the internal core, (5) analysis of enzymatic activity across the DE droplet populations *via* FACS. Right: FACS results for DE droplets containing *E. coli* expressing wide-type (wt) enzyme, unselected library (R0) and the library after one (R1), two (R2) and three (R3) rounds of sorting based on gate M1. Adapted with permission from ref. 54. Copyright 2006 Cell Press. (B) Schematic workflow of directed enzyme evolution using gel-shell beads (GSBs) and FACS. (1) Encapsulation of single *E. coli* cells expressing target enzyme in single emulsions and cell lysis to liberate the enzyme and its coding plasmid, (2) release of a fluorescent product by catalysis, (3) formation of GSB to entrap products, (4) high-throughput screening and sorting of GSB containing catalytically active hits *via* FACS, (5) recovery of variants with desired phenotypes, (6) iterative rounds of selection. Adapted with permission from ref. 93. Copyright 2014 Nature Publishing Group.

A. Aharoni *et al.*^54^ first reported using the DE-FACS technique and achieved high-throughput screening of enzyme libraries (see Fig. 3A). A variant gene library was transformed and cloned into *E. coli*, and the encoded protein, serum paraoxonase (PON1), was allowed to translate in the cytoplasm or on the surface of the *E. coli* cells. After encapsulation and cultivation of individual *E. coli* cells in DEs, compartments containing the fluorescent products, encapsulated cell variants, and the gene encoding enzyme were sorted by FACS. The isolated PON1 variants showed 100 fold improvements in TBLase catalytic efficiency. These works demonstrated the use of DE-FACS for directed enzyme evolution by directly monitoring the actual levels of endogenous cellular enzymes (rather expression levels of a reporter protein). Moreover, it shows the capability to identify rare phenotypes and genotypes for analysing large populations at a single-cell level.

To date, DE-FACS has been widely used for the directed evolution of different types of enzymes, including thiolactonase,^54^ β-glucosidase,^23^ arysulfatase^50^, G-type nerve agent hydrolase,^24^ protease,^22^ cutinase,^87^ cellulase,^88^ glucose oxidase,^89^ esterase,^90,91^ and polymerase,^92^ expressed in different microbial strains, such as *S. cerevisiae*, *E. coli* and *Bacillus subtilis* (*B. subtilis*). Moreover, hydrogel droplets with either core–shell (*e.g.*, agarose-alginate and polyacrylamide-agarose) or single emulsion (*e.g.*, PEG and alginate) structures have enabled the directed evolution of phosphotriesterase,^93^ hydrolytic enzymes^94^ for high-throughput cell cultivation and enzymatic analysis^95^ by integrating with FACS. For example, M. Fischlechner *et al.*^93^ utilised gel-shell microbeads with a polyelectrolyte shell to encapsulate an enzyme, its encoding DNA and fluorescent reaction product. FACS was able to identify active clones within the compartments at rates of higher than 10^7^ beads per hour (see Fig. 3B). This work allows the establishment of gel-shell microbeads as biomimetic compartments in which the content can be modified by directed evolution. The presence of the hydrogel shell allows more effective retention of target molecules (*e.g.*, cell-secreted molecules and cleaved products from the substrate) within the droplet compartments.

Besides the directed evolution of enzymes, microorganisms exhibiting high yields of other industrially essential metabolites, *e.g.*, organic acids and vitamins, have also been screened and selected using DE-FACS. J. M. Wagner *et al.*^96^ successfully isolated *Yarrowia lipolytica* (*Y. lipolytica*) mutants, resulting in a 54 fold increase in Riboflavin (vitamin B2) production parent strain. Since Riboflavin is an innate fluorescent product, no extra fluorescent substrates were used. Also, the isolated mutants can produce more extracellular products and the total amount of products than those separated by single-cell FACS, as confirmed by absorbance assays and high-performance liquid chromatography (HPLC). Similarly, X. D. Zhu *et al.*^97^ selected a *Bacillus coagulans* (*B. coagulans*) mutant yielding 52% higher lactic acid production than its parent strain. A pH-sensitive fluorescent sensor was adopted to indicate the level of lactic acid production inside the DEs, allowing the selection of DEs containing high-yielding mutants based on fluorescence.

DE-FACS has been widely used for directed molecular evolution by isolating single cells exhibiting a desirable phenotype. By combing with sequencing of the isolated variants, it provides valuable insights into the underlying mechanisms of molecular evolution. It is also obvious that this technology can be applied to other areas where analysing and sorting single cells in a high-throughput manner is needed, such as functional genomics and advanced protein engineering.

### 3.3 Single-cell detection

Sensitive and selective detection of single molecules (*e.g.*, DNA, RNA and proteins) and single cells are essential for probing cellular heterogeneity and discovering the unbiased diversity of cells in biology, chemistry and biomedicine. However, due to the limited initial numbers of single cells, traditional single-cell sequencing approaches are time-consuming and low-throughput. In contrast, droplet microfluidics is a promising technique for encapsulating and processing individual cells for whole-transcriptome or genomic analysis in a massively parallel manner with minimal reagent consumption. By taking advantage of FC (and FACS), ultra-high throughput and multi-parameter genetic analysis of a large heterogeneous population of cells at the single-cell level can be achieved to discover stochastic variations in a complex biological system.

**Fig. 4:**
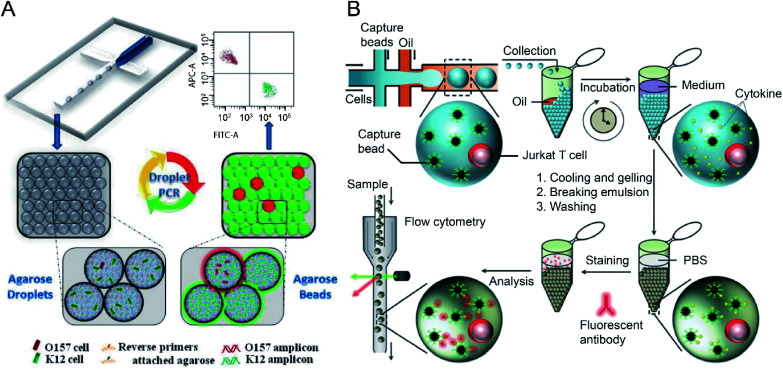
Droplet flow cytometry for single-cell detection. (A) Schematic diagram of rare pathogen detection by droplet-based PCR using agarose droplets and FACS. The rare pathogens (O157) and high-density normal bacteria (K12) are co-encapsulated into agarose droplets for PCR amplification. The PCR reagent mixture including two forwards primers labelled fluorescent dyes and two specific reverse primers specific for K12 and O157, respectively, were covalently conjugated to agarose. After PCR and cooling down, the agarose beads are analysed by flow cytometry for the detection of O157 cells. Adapted with permission from ref. 27. Copyright 2012 Royal Society of Chemistry. (B) Schematic workflow of the detection of cytokines secreted by single cells using agarose droplets and FC. Single Jurkat T cells were encapsulated within agarose droplets together with functionalized cytokine-capture nanobeads. After incubation, gelation, demulsification and washing, agarose beads were stained with fluorescent antibodies which can bind to the secreted cytokines, and high-throughput screening of cytokines secreted by single cells were performed by flow cytometry. Adapted with permission from ref. 25. Copyright 2013 Royal Society of Chemistry.

The integration of agarose microdroplets and FACS allows the identification and sorting of rare pathogens using polymerase chain reaction (PCR) signals from individual cells. A notorious pathogen causing many food-borne illnesses, *E. coli* O157:H7, was used as a target analyte, and the typical *E. coli* K12 was used as background noise.^27^ Massively parallel single-molecule PCR, singleplex and multiplex single-cell PCR were achieved in agarose droplets, and FACS determined the fluorescence of droplets (see Fig. 4A). This technique enabled the sensitive and quantitative analysis of single *E. coli* O157:H7 cells in the high background of 100 000 excess normal *E. coli* K12 cells. A similar work using agarose microdroplets for single-molecule Reverse Transcription Polymerase Chain Reaction (RT-PCR) was reported by H. Zhang *et al.*^28^ Agarose droplets demonstrated the capability to carry RT-PCR products after amplification at the single-molecule level. As a result, single-cell transcriptome analysis revealed a clear difference in the expression level of a cancer biomarker gene (*i.e.*, EpCAM) between different types of cancer cells (Kato III and MDA-MB-231 cells) at the single-cell level.

The detection of a single gene of individual microbial genomes from a mixture of microbial cells was achieved using hydrogel droplets with core–shell structures.^29^ Polyacrylamide droplets were used to encapsulate individual microbial cells and trap their genomes after cell lysis, which were further converted into agarose pico-reactors for subsequent multiple displacement amplification (MDA) reaction. After PCR, the polyacrylamide–agarose hydrogel droplets containing genomic DNA with target genes were fluorescently labelled with SYBR Green and analysed by FC. *E. coli* XL1 genomes containing a tetracycline resistance gene present 0.1% of *E. coli* MC1061 genomes without the tetracycline resistance gene can be differentiated.

The profiling of the cellular heterogeneity in cytokine secretion by cancer cells^12,98^ and immune cells^26^ at the single-cell level has been demonstrated. V. Turcanu *et al.*^30^ encapsulated lymphocytes in agarose microdroplets, which were functionalized with avidin to capture biotin-labelled cytokine-specific antibodies. Cytokines secreted by the encapsulated cells can be detected using a second fluorescent antibody specific to the cytokine. The analysis of several key cytokines, such as interleukin (IL)-4, transforming growth factor (TGF)-β, interferon-γ (IFN-γ) and IL-10 secreted by human peripheral blood mononuclear cells (PBMCs) and a rat keratinocyte cell line transfected with the human TGF-β gene was achieved. Also, polystyrene beads functionalized with specific antibodies have been used to capture cytokines secreted by immune cells entrapped in the droplet compartments. V. Chokkalingam *et al.*^25^ encapsulated single activated Jurkat Tet cells in monodisperse agarose droplets with functionalized cytokine-capture nanobeads. After incubation and phase transfer, the agarose beads were stained with fluorophore-labelled antibodies that bind to the cytokines, allowing flow cytometric detection of secreted cytokines (IL-2, IFN-γ, TNF-α) from single Jurkat T cells (see Fig. 4B). Besides hydrogel functionalisation and use of cytokine-capture nanobeads.

In addition to gene expression and cytokine, proteins,^98^ metabolites,^99^ and exosomes^100^ can be used as biomarkers for single-cell detection. For example, X. Yang *et al.*^101^ performed on-cell target detection and phenotypic probing of live cancer cells at the single-cell level by integrating droplet compartments with a fluorescence microscope. A biomarker-designated enzymatic fluorescent assay was carried out within the droplets, which was based on a DNA–antibody conjugate to the surface of live cancer cells. This approach shows great potential for the detection of different biomarkers at the single-cell level by modifying the antibody–DNA constructs with other antibodies of interest.

The single-cell agarose droplet flow cytometry method has tremendous implications in detecting rare pathogenic cells, analysing single-cell protein secretion and profiling of cellular heterogeneity at the single-molecule level and enumerating rare cancer cells, and shows great potential for numerous applications (*e.g.*, environmental science, drug discovery, diagnostics, cancer research, regenerative medicine, and synthetic biology).

### 3.4 Cell-to-cell interaction

Microbial interactions play a pivotal role in the dynamics and functions of microbial communities. However, it is difficult to reveal this intertwined phenomenon due to limitations in parallel culture and absolute abundance quantification of community members across environments and replicates.^102^ DFC has enabled rapid and quantitative investigation of the interaction networks between community members by encapsulating microbial cells into the micro-droplets that are screened and selected in parallel.

**Fig. 5:**
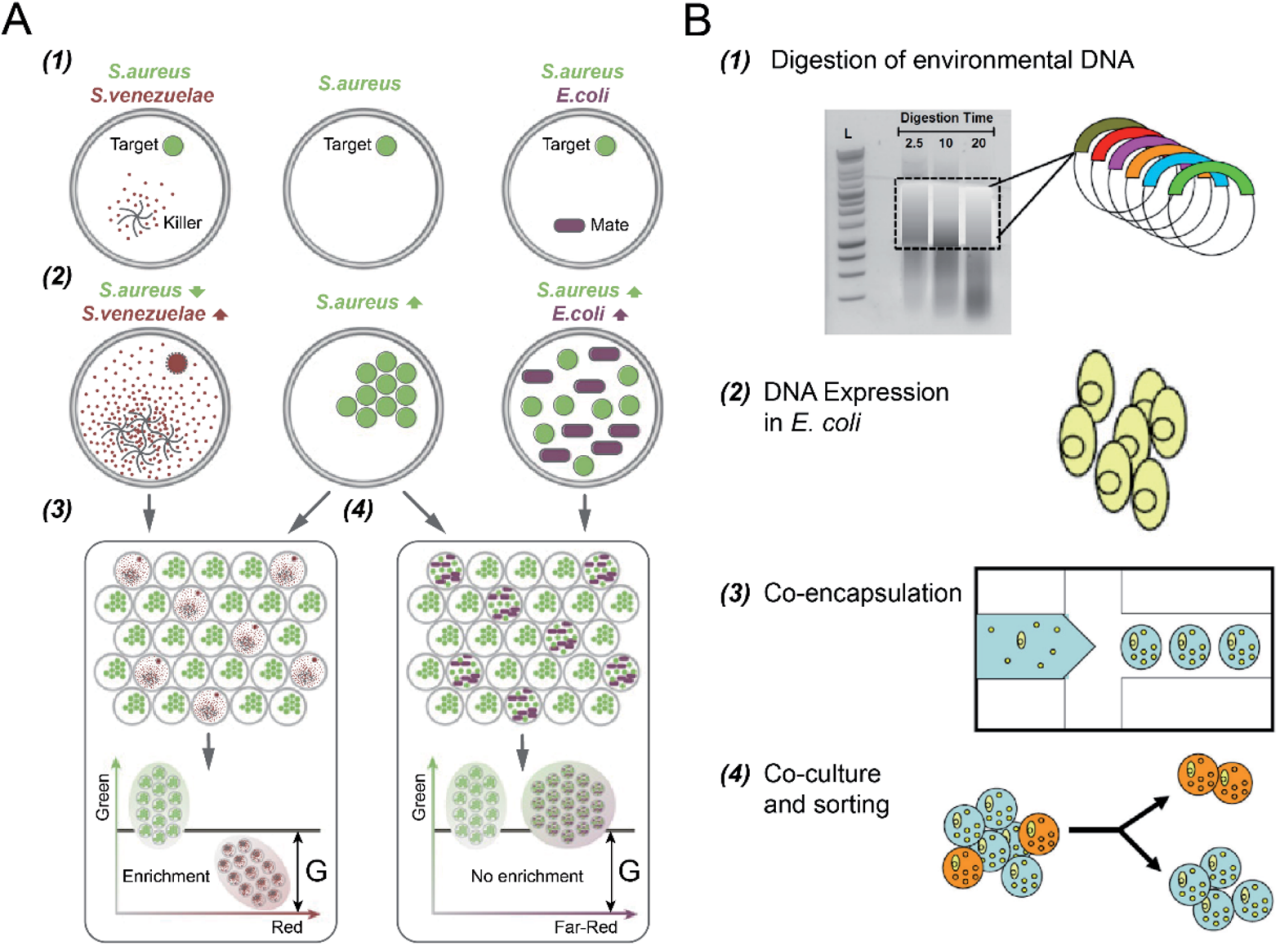
Droplet flow cytometry for cell-to-cell interaction. (A) Screening of bacteria inhibiting *S. aureus* growth using DE-FACS. (1) Target *S. aureus* cells with a GFP reporter were encapsulated with either antibiotic producer, *S. venezuelae* (secreting red fluorescent metabolites) or mate *E. coli* (with a far-red fluorescent reporter). (2) The inhibition of *S*. *aureus* with the growth of *S. venezuelae*, and the growth of both *E. coli* and *S. aureus*, which yielded different combinations of fluorescent signals. (3 and 4) Selection of DE droplets with the lowest fluorescence level caused the enrichment of killers *S. venezuelae* (3) rather than mates *E. coli* (4). Adapted with permission from ref. 32. Copyright 2017 National Academy of Sciences. (B) The workflow of high-throughput screening and selection of the co-culture of recombinant *E. coli* and *S. aureus* for antibiotic drug discovery *via* the integration of agarose droplets and FACS. (1) environmental DNA was subjected to a limited DNasel digestion, (2) metagenomic DNA was cloned and then transferred into *E. coli*, (3) individual recombinant *E. coli* were co-encapsulated with live *S. aureus* (small yellow spheres), (4) microdroplets were labelled with a fluorogenic viability probe and then selected by FACS to isolate *E. coli* secreting bacterial natural products. Adapted with permission from ref. 33. Copyright 2013 John Wiley & Sons, Inc.

S. S. Terekhov *et al.*^32^ investigated different types of bacterial cell-to-cell interactions using DE-FACS (see Fig. 5A). A common pathogen, *Staphylococcus aureus* (*S. aureus*), was co-cultured with a mate (*E. coli*), which led to the growth of both bacteria in droplets, and an antibiotic producer, *Streptomyces venezuelae* (*S. venezuelae*), which inhibited the growth of *S. aureus* as a killer. Since all the bacteria used in the experiment have different fluorescence reporters, killers and mates can be distinguished, and droplets encapsulating with killers can be selected. This technique was then used to identify rare *S. aureus* killers from human oral microbiota by selecting the droplets containing a high concentration of killers and a low concentration of *S. aureus*. Slow-growing oral microbiota species that inhibit the growth of *S. aureus* were isolated, and genera associating with inhibitory activity was revealed. Using the same platform, S. S. Terekhov *et al.*^31^ performed environmental microbiota communities profiling (*i.e.*, a Siberian bear oral microbial community) to discover secondary microbial metabolites. The target pathogen, *S. aureus*, was co-encapsulated with individual cells from the heterogeneous community to identify rare antimicrobial strains. As a result, bacterial clones with inhibitory activity, especially ‘unculturable bacteria’ that cannot be recognized by standard bulk methods, were selected and identified.

In addition to DEs, hydrogel (agarose) microdroplets have also been used to uncover yeast–bacterium interactions for antibiotic drug discovery,^33^ where bacterial pathogens were co-cultured with recombinant host microorganisms capable of secreting biocatalytic antibiotics and/or secondary metabolites (see Fig. 5B). *S. cerevisiae* strains, including both the positive control (with the secretion of bacteriolytic enzyme lysostaphin) and the negative control (with the secretion of inactive lytic hydrolase enzyme), were co-encapsulated with a target pathogen *S. aureus*, respectively. Following host *S. cerevisiae* growth and protein induction, agarose microdroplets were stained with a fluorogenic viability probe (SyTox Orange) that selectively stains killed target pathogens, and then analysed by FACS. Rare positive clones producing a lytic hydrolase specific for *S. aureus* can be isolated with an enrichment factor of up to 10 000 fold relative to the initial mixture.

Moreover, the interactions between microalgal and bacterial cells have been investigated. J. Ohan *et al.*^34^ utilised agarose gel droplets to co-encapsulate individual bacterial cells from a complex microbial community with *Chlorella sorokiniana* (*C. sorokiniana*), a candidate algal strain for biofuel production. Using FACS, viable bacteria species exhibiting the desired phenotype (*i.e.*, improved algal growth rate) were isolated and recovered. Researchers have also reported the use of DFC for the screening of co-cultures of mammalian cells and yeast cells. Individual yeast secretors (*e.g.*, *S. cerevisiae*^103^ and *Pichia pastoris*^104^) and mammalian reporter cells were co-encapsulated and cultivated in agarose microdroplets, which is followed by FACS to select clones secreting bioactive recombinant proteins. These studies demonstrated the feasibility of DFC for screening and isolation of protein variants produced by microbes, which would enable and accelerate the discovery of new functional biologics, such as cytokines, antibodies, and soluble receptors.

Using the same technique, E. Tumarkin *et al.*^105^ uncovered mammalian cell interaction networks. MBA2 cell line (secreting interleukin-3 (IL-3)) and the human megakaryoblastic leukemia cell line, M07e (depending on IL-3 for proliferation and survival), were co-encapsulated within agarose hydrogel droplets at different ratios. Flow cytometric analysis results showed that local paracrine secretion could modulate the viability of the factor-dependent M07e cells. Besides, IL-3 secreting MBA2 cells were co-encapsulated with umbilical cord blood (UCB) cells to determine the impact of IL-3 on a heterogeneous cell population, which revealed subpopulations that are primarily dependent on locally delivered IL-3 (*i.e.*, CD14 + cells).

Mammalian cell-to-cell interactions are crucial in cell development, tissue and organ homeostasis, and immune interactions in disease.^106,107^ Researchers have achieved *in vitro* co-culture of different types of mammalian cells to investigate the underlying metabolic processes and interactions.^108^ Unfortunately, despite the biological and biomedical significance of mammalian cellular interactions, there are still quite limited reports in literature regarding the use of the DFC in the study of mammalian cell communications. With further advances in droplet compartments and cell co-culture models, we can expect that wider communities will adopt DFC for cell-to-cell interaction analysis.

## 4. Applications

By pairing FC with emulsion droplets (*i.e.*, W/O/W DE and hydrogel droplets), it enables the screening and isolation of microdroplets encapsulated with rare cells exhibiting properties of interest and characterization of unexpected cell-to-cell heterogeneity within the population. Table 1 summarised the capabilities of DFC for high-throughput single-cell analysis. This integrated technology is critical for various applications, including but not limited to drug screening, medical diagnosis, metabolic engineering and cell biology.

**Table 1:** Summary of capabilities of droplet flow cytometry for high-throughput single-cell analysis

Microdroplet	Target cell	Application
**Single-cell cultivation**		
W/O/W	*E. coli*	Cell culture (from proliferation to death)^56^
W/O/W	Oral microbiota of Siberian bear	Cell culture (with antibiotics)^31^
	Human fecal microbiota from patient and healthy donors	
W/O/W	Mouse embryonic stem cells (E14), macrophage cells (LM-1) and embryonic fibroblasts (NIH 3T3); human T lymphocytes (Jurkat) and embryonic kidney cells (HEK 239T)	Screening of cell encapsulation ratios for different types of cells^18^
Gelatin	*E. gracilis*, *C. reinhardtii*	Chlorophyll and lipid accumulation^79^
Agarose	*E. coli*	MIC of rifampicin determination; mutant isolation^84^

**Molecular evolution**		
W/O/W	*E. coli*	Thiolactonases (100 fold increase)^54^
		Arysulfatase^50^
		β-Glucosidase (∼2 fold increase for lactose)^23^
		G-type nerve agent hydrolase (10^4^-fold increase)^24^
		Cutinase (8 fold increase)^87^
		Esterase (2 fold increase)^90^
		Polymerase (1200 fold increase)^92^
W/O/W	*B. subtilis*	Protease (1.6 fold increase)^22^
W/O/W	*S. cerevisiae*	Endoglucanase-II cellulase (20 fold increase)^88^
		Glucose oxidase (5.8 fold increase)^89^
Agarose-alginate polyelectrolyte	*E. coli*	Phosphotriesterase (20 fold increase)^93^
PEG	*E. coli*	Hydrolytic enzyme (3 fold increase)^94^
W/O/W	*Y. lipolytica*	Riboflavin (54 fold increase)^96^
W/O/W	*B. coagulans*	Lactic acid (52% more effective)^97^

**Single-cell detection**		
Agarose	*E. coli* (O175:H7 and K12)	Detection of O157:H7 (0.0001%)^27^
Agarose	Human gastric carcinoma cells (Kato III) and breast cancer cells (MDA-MB-231)	Differentiation in gene expression level of a cancer biomarker (EpCAM)^28^
Polyacrylamide-agarose	*E. coli* XL1	Differentiation of a tetracycline resistance gene^29^
Agarose	*T lymphocytes*	Secretion of cytokines: IFN-γ, IL-4, IL-10 or TGF-β^30^
		Secretion of cytokines: IL-2, IFN-γ, TNF-α^25^

**Cell-to-cell interaction**		
W/O/W	*S. aureu* + microbes	Antibiotic producer discovery^32^
	*S. aureus* + *S. venezuelae*	
	*S. aureus* + *E. coli*	
W/O/W	Oral microbiota of Siberian bear + *S. aureus*	Antibiotic Ami discovery^31^
Agarose	*S. cerevisiae* + *S. aureus*	Lytic hydrolase producer for *S. aureus*^33^
Agarose	*C. sorokiniana* + *Pseudomonas* spp.	Viable bacteria improving algal growth^34^
Agarose	Stromal cells (MBA2) + leukemia cells (M07e)	Impacts on M07e cells survival^105^
Agarose	*S. cerevisiae* + murine Ba/F3 reporter cells	Cytokine secretion by *S. cerevisiae* libraries^103^
Agarose	*P. pastoris* + cancer cells (A431)	Antibody secretion by *P. pastoris* libraries^104^

### 4.1 Drug discovery

Due to the emergence and spread of drug resistance and new diseases, there is an urgent need for new drug development. An effective technique for high-throughput drug screening plays a critical role in finding a new drug against the target of particular diseases, where diverse compound libraries are tested under different conditions in parallel with the drug target.^109^ Cell-based assays are always involved in this process, which provides substantive information on various cellular responses upon compound exposure, reducing the need for animal models. DFC allows high-throughput monitoring and screening of cultures of isolated single cells (*e.g.*, bacteria cells) within droplet compartments in response to a specific drug or a combination of multiple drugs. This reveals a heterogenic reaction that cannot be obtained by traditional drug screening methods (which are based on the average of the entire population). In addition, the use of emulsion droplets enables the analysis of a single cell's anti-drug response (*e.g.*, antibiotic resistance^31,32,84^). Microdroplets are also used to analyse the drug response of a cancer cell line with different phenotypes, showing a clear distinction in drug response as a function of a specific phenotype. This ability to reveal different cell line responses to the same compound allows scientists to gain important information for new drug discovery and development.

### 4.2 Medical diagnosis

The presence and the stage of a particular disease can be indicated by sensitive and reliable detection of specific biomarkers, bacteria and viruses. For example, the proteins secreted by cancer cells (*i.e.*, secretomes) are a fundamental source of biomarkers.^98^ Although conventional immunoassays, in particular, enzyme-linked immunosorbent assay (ELISA)^110,111^ have been widely used to quantify protein biomarkers in bulk, the detectable concentrations of proteins are limited to generally above the picomolar range, making them unable to detect cancer biomarkers that exist in biological fluids at concentrations in the range of 10^−12^ to 10^−16^ M.^112^ Moreover, it is quite challenging to perform characterisation of immune responses in many cases, as it requires the isolation of individual immune cells. For instance, a diversity of T cells can be produced by human immune system, but only a fraction of them are capable of being recognised and distinguished from those infected and mutant cells. Therefore, DFC has been applied to detect low-abundance proteins secreted from single cells and to quantify single-cell immune signatures.^113^ Tumour heterogeneity can confound the detection and diagnosis of cancer patients, which has led to high-throughput single-cell analysis platforms to aid in the determination of cancer malignancy.^114–117^ Also, co-encapsulation of distinct cell types in microdroplets holds great potential for future cancer research by probing heterogeneous cell–cell interactions (*e.g.*, cancer-immune cell interactions in cancer surveillance).

### 4.3 Metabolic engineering

Due to the limited knowledge of complicated cellular networks, directed evolution has played an essential role in improving the performance and functionality of industrially important microbes.^118^ High-throughput and cost-effective screening tools are required in the selection of super variants. Emulsion droplets provide compartmentalization of individual cells from a massive variant library containing genetic diversity. Each cell-laden droplet in aqueous suspension can be considered a distinct event in the cytometers. Selection of improved variants from the library can be carried out based on the level of phenotypes (*e.g.*, secreted protein production), allowing to establish the linkages of phenotypes and genotypes for directed evolution of functional proteins. This mimics the process of natural evolution in a laboratory setting, which relies on iterative cycles of genetic diversity generation and phenotypic selection to isolate evolved industrial strains with desired traits. DFC has been providing an efficient screening avenue to improve microbial strains for enhanced enzymatic activity, and improved production of value-added bioproducts (*e.g.*, vitamins and organic acids).^96^

### 4.4 Cell biology

DFC opens new opportunities in cell biology by improving our understanding of how the behaviours of individual cells shape biological processes. Microbial cells from a complex community can be randomly encapsulated into microdroplets that can be used as miniaturized compartments and screened or sorted in parallel by flow cytometers to study how the community members interact. This high-throughput approach can rapidly resolve microbial interaction networks across different initial community states, population sizes, and environments, enabling a better understanding of the critical parameters shaping the structures and functions of microbial communities.^102^ For example, co-encapsulation of two bacterial strains allows for discrimination between viable and non-viable cells, identifying the conditions that support the growth of unculturable cells and improved understanding of mechanisms underlying bacterial inhibitory activity.^31^ Moreover, co-culture of a microalgal strain with a pool of environmentally sourced bacterial cells allows the isolation of the ones that can induce a desired phenotype (*i.e.*, improvement in algae growth rate).^34^

## 5. Current challenges and perspectives

As discussed above, the ability to screen and select microdroplets encapsulated with single cells by FC brings in new possibilities in various fields. Compared with traditional well plate assays, this integrated technique has impressive advantages, such as high throughput and low cost. However, there are still issues that limit DFC as a common technique for high-throughput single-cell analysis.

### 5.1 Molecule leakage

The first area that needs improvement is the transport and leakage of the molecules encapsulated within emulsion droplets. To ensure accurate and effective in-drop assays, it is essential to maintain all the contents within the droplet compartments during the process (*e.g.*, incubation, relocation and manipulation) without any cross-contamination or analyte loss.^22^ Unfortunately, there is a possible leakage of small molecules from the droplets into the surrounding oil and neighbouring droplets, which may be due to the hydrophobicity and solubility of fluorophores, formation, and transport micellular or vascular structures, or media pH.^50^ This results in the reduction of sensitivity and resolution of biomechanical assays performed in microdroplets.

Although several approaches have been reported to increase the containment of small molecules within droplets, such as the use of fluorinated oils,^119,120^ decrease in the concentration of surfactants,^121^ and addition of bovine serum albumin (BSA) or sugar additives into the inner aqueous phase,^122^ some fluorophores of widely used substrates are hydrophobic and suffer rapid diffusion or exchange between droplets. One interesting approach is nanoparticles that can interface with target hydrophobic molecules to prevent their leakage.^123^

Another approach that may circumvent this limitation is the chemical or enzymatic modification of the hydrophobic products into hydrophilic, charged molecules that cannot diffuse into the oil shell.^55^ There is still a need for further development of new fluorescence dyes and metabolic biomarkers that can be confined within the droplets and new biochemical technologies that can prevent the leakage of small molecules from droplets.

### 5.2 Access to droplet microfluidic technology

The second important challenge is the restricted access to droplet microfluidic technology. Currently, microfluidic systems are mainly designed, fabricated and operated by specialized engineering laboratories, and the applications in life and biomedical sciences achieved are based on the collaborations with engineering research groups. Therefore, more efforts should be put into commercialising simple and cost-effective instruments and surfactants to facilitate wider adoptions, allowing users without expertise in microfluidics to carry out the wide range of applications summarised above.

We note that a few examples of droplet microfluidic technology have been successfully commercialized, *e.g.*, DropSeq and InDrops by 10× Genomics, which enables single-molecule detection by measuring thousands of independent amplification events within a single sample. Moreover, there are many companies provide a wide catalogue of standard microfluidic chips for various applications, and help with design, prototyping and fabrication of full custom microfluidic chips that meet specific needs. It would be helpful to push some relatively basic instruments for individual applications (*e.g.*, enzymatic assays and PCR) into the market. Also, it looks great to make the technology instruments capable of integration with the multi-well plate format, so that highly parallelized downstream procedures can be carried out with existing liquid handling robots. We foresee that the accessibility of basic microfluidic technology will be expanded to non-specialists in the broader research and clinical communities.

### 5.3 Optical interrogation

Another challenge is that the screening and analysis of droplet bioreactors have mainly relied on the detection of fluorescent products. Although fluorescent labelling allows multiple readouts simultaneously with high sensitivity, it is often challenging to incorporate them into biochemical assays. The native target is not fluorescently active, and adding a fluorescent dye may cause cellular cytotoxicity, photobleaching, and interface with cellular metabolism.^124^ Also, there are limited fluorescent dyes available to probe complex cell activities in microdroplets. Therefore, developing a label-free optical detection technology for sensitive detection and characterization of droplet bioreactors would be of great promise by expanding the available assays in droplet screening and sorting.

Different optical techniques have been used to analyse droplets beyond fluorescence detection. For example, absorbance and mass-based detection^41,125^ has been used for enzymatic activities in droplets, but it suffers from relatively low specificity. Although Raman spectroscopy has been applied to quantify single-cell metabolite and enzyme production in microdroplets,^42,126^ the relatively high level of noise and low sensitivity limit its comprehensive implementation in high-throughput assays. We note that recent advances in coherent Raman spectroscopy^127–130^ have enabled high-throughput (*i.e.*, 2 kHz) and label-free analysis of heterogeneous populations at the single-cell level, which holds great promise for high-accuracy cell classification.

Moreover, imaging flow cytometry (IFC) has become an attractive tool for single-cell analysis in diverse biomedical fields, and a two-dimensional image signal has been employed for real-time classification and sorting of droplets encapsulating with cancer^131,132^ and planktonic cells.^133^ Combining with high-speed image-based sorting,^134–139^ this will eventually be used in the process of isolating individual cells exhibiting properties of interest. With further advances in sensitivity and specificity of label-free optical detection techniques, we expect that droplet encapsulation and compartmentalization will expand into a more versatile platform technology for high-throughput single-cell analysis.

## 6. Conclusions

The ability to encapsulate, cultivate, monitor and select single cells in a large number of droplet bioreactors provides new opportunities in tackling numerous problems in fundamental and applied biology and health. New toolsets and new fluorescent toolsets may deliver breakthroughs in tackling the challenges regarding transport and leakage of small molecules in droplets. The availability of droplet technology has a chance to increase *via* collaborations between engineers and biologists rapidly by commercializing standard instruments and chemicals (*e.g.*, surfactants) for droplet generation and manipulation. We envision that further development of droplet systems and optical detection techniques should allow DFC to expand into a more typical and versatile platform technology for high-throughput single-cell analysis.

## Conflicts of interest

There are no conflicts to declare.

## Supplementary Material
